# The Lutein and Zeaxanthin in Pregnancy (L-ZIP) study—carotenoid supplementation during pregnancy: ocular and systemic effects—study protocol for a randomized controlled trial

**DOI:** 10.1186/s13063-021-05244-2

**Published:** 2021-04-22

**Authors:** Emmanuel Kofi Addo, Aruna Gorusupudi, Susan Allman, Paul S. Bernstein

**Affiliations:** 1 Department of Ophthalmology and Visual Sciences, John A. Moran Eye Center, University of Utah School of Medicine, 65 Mario Capecchi Drive, Salt Lake City, UT 84132 USA; 2 Department of Nutrition and Integrative Physiology, University of Utah, Salt Lake City, UT USA

**Keywords:** Carotenoids, Supplementation, Maternal, Pregnancy, Prenatal, Visual function, Biomarkers, Infants, Lutein and zeaxanthin, Randomized controlled trial

## Abstract

**Background:**

Lutein (L), zeaxanthin (Z), and *meso*-zeaxanthin (MZ), collectively called the macular pigment (MP), are dietary carotenoids that preferentially localize in the macula of the human eye. MP protects the macula from photo-oxidative damage and enhances visual function. Inadequate maternal intake of carotenoids, coupled with the placental transfer of maternal carotenoids to support fetal brain and retina development, potentially put mothers at risk of depletion systemically and in their ocular tissues. Presently, maternal carotenoid status throughout pregnancy remains poorly characterized, and no prospective randomized controlled trial of L and Z supplementation via prenatal vitamins has assessed maternal and infants’ systemic and ocular carotenoid status during pregnancy. We hypothesize that prenatal maternal carotenoid supplementation will counteract maternal carotenoid depletion during pregnancy and will improve biomarkers of carotenoid status of both mothers and infants.

**Methods:**

Lutein and Zeaxanthin in Pregnancy (L-ZIP) is a phase 2, single-center, prospective, double-masked, randomized active-controlled clinical trial conducted at the John A. Moran Eye Center, University of Utah, Salt Lake City, UT, USA. Participants consume a daily standard prenatal multivitamin with no added carotenoids and are randomized (1:1 allocation) to receive either a capsule containing 10 mg L and 2 mg Z in safflower oil (Carotenoid group) or a capsule containing only safflower oil with no added carotenoids (Control group) for a period of 6 to 8 months. Skin, serum, and ocular carotenoids are measured at every study visit (i.e., within the first trimester [baseline], second trimester, third trimester, and 0–2 weeks postpartum). Skin carotenoid assessment is by resonance Raman spectroscopy (RRS); serum carotenoid status is quantified using high-performance liquid chromatography (HPLC); and MP is measured with the dual-wavelength autofluorescence. Infants’ MP and foveal anatomy are assessed using RetCam retinal camera and Bioptigen SD-OCT, respectively. The primary outcomes are changes in maternal systemic and ocular carotenoid status during pregnancy.

**Discussion:**

L-ZIP is the first prospective RCT to investigate maternal carotenoid status throughout pregnancy and to determine whether prenatal maternal carotenoid supplementation will offset maternal carotenoid depletion and improve biomarkers of maternal and infant’s carotenoid status. Findings from L-ZIP will strengthen recommendations regarding prenatal carotenoid supplementation and consequently inform policy decisions.

**Trial registration:**

ClinicalTrials.gov NCT03750968. Registered on November 23, 2018

## Administrative information

The order of the items has been modified to group similar items (see http://www.equator-network.org/reporting-guidelines/spirit-2013-statement-defining-standard-protocol-items-for-clinical-trials/).

**Table:** 

**Title {1}**	The Lutein and Zeaxanthin in Pregnancy (L-ZIP) Study – Carotenoid Supplementation During Pregnancy: Ocular and Systemic Effects – Study Protocol for a Randomized Controlled Trial
**Trial registration {2a and 2b}**	ClinicalTrials.gov identifier: NCT 03750968 Registered on November 23, 2018
**Protocol version {3}**	IRB_00116610 / Version 4.0 / 09 Oct 2019
**Funding {4}**	NIH Grants EY029857 and EY014800, Research to Prevent Blindness
**Author details {5a}**	^1^Department of Ophthalmology, John A. Moran Eye Center, University of Utah Health, Salt Lake City, Utah, USA ^2^ Department of Nutrition and Integrative Physiology, University of Utah, Salt Lake City, Utah, USA
**Name and contact information for the trial sponsor {5b}**	National Institutes of Health (NIH), 9000 Rockville Pike, Bethesda, Maryland 20892, USA
**Role of sponsor {5c}**	The funding sources had no role in the study design; collection, analysis, and interpretation of data; writing of the report; or the decision to submit the report for publication.

## Introduction

### Background and rationale {6a}

Carotenoids are naturally occurring lipophilic isoprenoid pigments synthesized mainly in plants and some microorganisms [1]. Thus far, about 750 carotenoids exist in nature, with approximately 50 detectable in the human diet. The human serum has about 15–20 carotenoids, with only three, lutein (L), zeaxanthin (Z), and *meso*-zeaxanthin (MZ), preferentially localized in the macula of the human eye, an area specialized for central and distinct spatial vision [2, 3]. L, Z, and MZ are collectively called the macular pigment (MP) and account for the yellow spot appearance of the *macula lutea* [4]. MP protects the macula from photo-induced oxidative damage through its blue light filtering property, antioxidant, and anti-inflammatory activity [3, 5–8]. In clinical studies, MP enhances visual and cognitive function and serves as preventive, therapeutic agents for retinal pathologies, such as age-related macular degeneration (AMD), the leading cause of irreversible blindness in developed countries [9–17]. Of note, carotenoids are not synthesized de novo in animals, and as such, must be obtained exclusively through the diet (primarily green leafy vegetables and yellow/orange fruits and vegetables rich in L and Z) or by supplementation [6, 18–20]. MZ is not commonly found in nature and is synthesized enzymatically in the retinal pigment epithelium from L [21–23].

Recent evidence indicates that MP accumulates in the retina even before birth and may enhance visual function [24–28]. Notably, L and Z are the most abundant carotenoids in the placenta, and placental L and Z significantly correlate with umbilical cord blood and maternal serum L and Z [29]. Also, L and Z accumulate in all brain lobes of infants associated with cognition, suggesting a possible role in infant cognitive development [30, 31]. Moreover, previous studies in our laboratory have shown detectable levels of MP in infants right after birth, which significantly correlate with maternal serum zeaxanthin levels [32]. MP continues to accumulate in the retina, as evidenced by gradual increases from birth until 7 years of age [33]. These findings imply that macular carotenoids may play a crucial physiological and protective function throughout the lifecycle and may be particularly important during infant visual development.

A growing body of literature supports the notion that maternal dietary intakes significantly impact mother’s carotenoid levels, determine pregnancy outcome, and affect fetal health and development [34, 35]. Hence, newborns’ carotenoid status is largely dependent on maternal nutrition. Of note, carotenoid ingestion among pregnant women varies across populations [36]. Thus, pregnant women who smoke and are younger tend to consume lower amounts of carotenoids [35, 37, 38]. The Food and Drug Administration (FDA) regards carotenoids as Generally Recognized As Safe (GRAS) nutrients for human ingestion, as they pose little to no adverse effect on health even at doses much higher than the 10–20 mg per day commonly found in many over-the-counter supplements. However, Americans generally consume low amounts of carotenoids (1–2 mg/day of L and about 0.2 mg/day of Z) [39]. Moreover, it is worth mentioning that pregnancy presents with oxidative stress, which places extreme demand on the body’s antioxidants such as carotenoids [40–42]. Also, fetal growth and development rapidly occur during the third trimester of pregnancy, specifically the fetus’s central nervous system and retina. This fetal developmental phase warrants the transfer of large amounts of nutrients such as docosahexaenoic acid (DHA) and carotenoids from the mothers to the fetus through the placenta [43]. The increased fetal demand for these nutrients for growth and development potentially puts mothers at risk of ocular and systemic carotenoid depletion, affecting maternal visual function and possibly increasing the risk of having AMD, a later in life disease which is more prevalent in women.

Globally, prenatal micronutrient supplementation is the standard-of-care for expectant mothers, but only a few prenatal micronutrients such as folate and DHA are backed by prospective clinical research [44–47]. Prenatal supplements containing L or Z were nonexistent until Abbott Nutrition introduced Similac Prenatal Vitamins containing 6 mg of L into the American market in 2013 in the hope that it would improve infant neural and visual development. In theory, the claim was biologically plausible, but the absence of a supportive prospective clinical data resulted in low market penetration and subsequent withdrawal of the product from the market a few years later.

Despite the conflicting reports on maternal carotenoid status during pregnancy [32, 48–50], no clinical trial to date has characterized maternal macular pigment and skin levels throughout pregnancy. Also, given the low consumption of carotenoids and increased maternal risk of carotenoid depletion, supplementing mothers with carotenoids during pregnancy may prevent carotenoid depletion and enhance both mothers’ and infants’ visual function and biomarkers of carotenoid status.

### Objectives {7}

We hypothesize that maternal carotenoid supplementation during pregnancy will counteract maternal carotenoid depletion in the macula, skin, and serum during pregnancy (primary hypothesis). Our secondary hypothesis will test whether carotenoid supplementation during pregnancy increases mother and child’s macular pigment, serum, and skin carotenoids at birth. Finally, our exploratory hypotheses will investigate whether infants with higher MP levels have more mature foveal architecture than infants with lower MP levels and whether supplemented mothers have superior visual function than unsupplemented mothers at the end of pregnancy.

### Trial design {8}

**Fig. 1:**
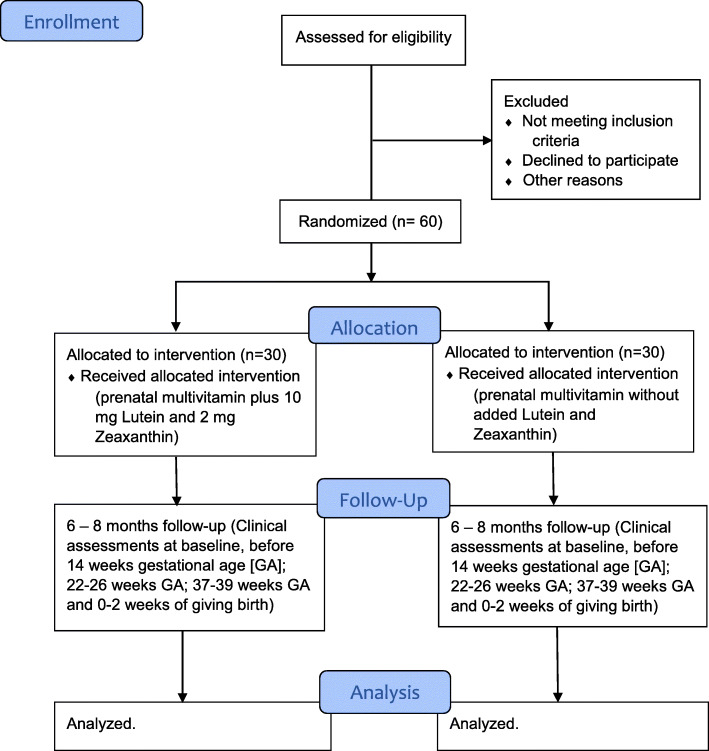
Lutein and Zeaxanthin in Pregnancy (L-ZIP) trial consolidated statement of reporting trials flow diagram

This study is a phase 2, single-center, prospective, randomized, active-controlled, double-masked clinical trial. The study will last for 2 to 3 years. Participants will be randomly assigned (1:1 allocation) to receive either prenatal vitamins plus 10 mg lutein and 2 mg zeaxanthin (the Carotenoid group) or prenatal vitamins without 10 mg lutein and 2 mg zeaxanthin (the Control group) to be taken daily until delivery of the baby. Of note, each participant’s one eye will be designated as the study eye for purposes of macular pigment measurements (typically the non-dominant eye unless the physician or patient decides otherwise). Figure 1 shows the flowchart illustrating participant’s enrollment, random assignment, follow-up, and analysis for the Lutein and Zeaxanthin in Pregnancy (L-ZIP) study.

## Methods: participants, interventions, and outcomes

### Study setting {9}

This study is a single-center study conducted at the John A. Moran Eye Center, the University of Utah, Salt Lake City, UT, USA. Participants’ recruitment will start during their first-trimester prenatal visit (before week 14) to the obstetrics and gynecology clinics at the University of Utah Hospital.

### Eligibility criteria {10}

#### Inclusion criteria

*The inclusion criteria are:*

1. Pregnant women of all races and ethnicities with uncomplicated obstetric histories.
2. Pregnant women aged 18 years or older who plan to deliver at the University of Utah Hospital and capable of providing informed consent.
3. Women who intend to deliver by either vaginal or Caesarian section.

#### Exclusion criteria

*Women will be excluded if they:*

1. Have regularly (i.e., daily for the past 6 months) taken carotenoid supplements with more than 0.5 mg of lutein and/or zeaxanthin.
2. Have significant eye disease associated with macular pigment abnormalities such as Stargardt disease, albinism, or macular telangiectasia type II (MacTel).
3. Have any conditions associated with high-risk pregnancies such as adolescent pregnancy, multifetal pregnancy, current or history of diabetes, pre-eclampsia, previous premature delivery, drug abuse, or other significant medical illness.

### Who will take informed consent? {26a}

All participants must provide written informed consent in compliance with International Conference on Harmonization (ICH) Guidance E6 before enrollment into L-ZIP. The Research Ethics Committee and the Institutional Review Board (IRB) of the University of Utah, Salt Lake City, UT, granted ethical approval for the L-ZIP study. L-ZIP will adhere to the tenets of the Declaration of Helsinki, the ICH Harmonized Tripartite Guidance for Good Clinical Practice (IHC-GCP E6 [R1]), and will fully comply with the code of ethics regarding participant enrollment, study assessment, and data protection.

The Principal Investigator (PI) or a designee trained in consenting obtains written informed consent from each participant. The study team will communicate the study’s purpose, the assessment procedures involved, and the potential hazards to the participants in non-technical terms. The participant will have ample time to consider the study’s implications before deciding to participate in the study. The participant and/or legal guardian will be required to sign and date an informed consent form (ICF). Enrolled participants may withdraw from the study at any time without jeopardizing their medical care. The PI will retain the original, signed ICF for study participation in the subject’s medical record and will provide the subject and/or legal guardian with a copy of the signed consent form.

Of note, if there are any changes/amendments to the approved protocol which may directly affect the subject’s decision to continue participation in the study, the ICF will be amended to incorporate the changes to the protocol, and the participant will be required to re-sign the IRB-approved amended ICF.

### Additional consent provisions for collection and use of participant data and biological specimens {26b}

On the consent form, the research team seeks participants’ permission to share relevant data with regulatory authorities and IRB, where appropriate.

## Interventions

### Explanation for the choice of comparators {6b}

L-ZIP is an active-controlled trial, so it will be unethical to deny mothers prenatal vitamins (standard-of-care for pregnant women globally). Therefore, mothers in the control group only received prenatal vitamins plus a softgel with no active study supplement (10 mg lutein and 2 mg zeaxanthin). Also, to ensure that both study coordinators and participants are oblivious of the group to which participants are randomized, softgels with similar external appearance but different compositions are used for the study. It is worth pointing out that the active study supplement exceeds most Americans’ dietary intake (1–2 mg of L and about 0.2 mg of Z per day). However, they are comparable to levels known and reported to be safe in the Age-Related Eye Disease Study (AREDS) 2 formulation [14, 39]. Details of ocular and systemic analysis of carotenoid status have been provided in the “Plans for assessment and collection of outcomes {18a}” section.

### Intervention description {11a}

Eligible participants who provide informed written consent are randomized in a ratio of 1:1 to the two arms of the study (the Carotenoid or the Control group). The study team instructs participants in the Carotenoid group to take one each of the Spring Valley™ Prenatal Multi + DHA capsule (or equivalent product) and a capsule containing 10 mg lutein and 2 mg zeaxanthin in safflower oil (commercially available as Lutein-2 mg Zeaxanthin Softgels, prepared and provided by Kemin Health, Des Moines, IA, USA). Likewise, the study team advises participants in the Control group to take one of the Spring Valley™ Prenatal Multi + DHA capsules (or equivalent product) and a softgel containing only safflower oil with no added carotenoids (prepared and provided by Kemin Health, Des Moines, IA, USA).

Baseline measurements of each participant’s carotenoid status (in the skin, serum, and eye) and visual function will be performed at the enrollment time before dispensing study supplements. Repeated measurements of the participant’s carotenoid status and visual function will be obtained at 22–26 weeks GA and again at 37–39 weeks GA. All participants will undergo a final study assessment within 2 weeks of infant delivery.

Assessment of infant carotenoid status includes obtaining a serum sample collected from the cord blood at the delivery time and portions of the placenta. Other ocular and skin evaluations will be performed either in the Well Baby Nursery at the University of Utah or within the first 2 weeks of life at a routine postnatal outpatient clinic visit.

### Criteria for discontinuing or modifying allocated interventions {11b}

The prenatal supplements used in this study are within the standard-of-care for supplements prescribed to women during pregnancy. The FDA lists the active study supplements (lutein and zeaxanthin) as GRAS for use in prenatal supplements, so we expect no systemic toxicity. Nevertheless, participants may withdraw from the study at any time for any reason. Also, the PI reserves the right to withdraw a patient from the study in the event of an intercurrent illness, adverse event, non-compliance with study supplement use, or other reasons.

### Strategies to improve adherence to interventions {11c}

Enrolled participants’ follow-up visits are planned to coincide with routine prenatal visits with their obstetrician, at approximately 22–26 weeks GA, at 37–39 weeks GA, and within 2 weeks of giving birth. The study coordinator gives all participants a tracking log to document supplement use. Prior to a follow-up appointment, the study team contact participants over the phone or through text messages to remind participants to regularly take the study intervention and bring their supplement bottles for pill counting. Finally, participants receive reimbursement for their time and transportation in the form of a gift card. These measures are to ensure that participants comply with the study intervention.

### Relevant concomitant care permitted or prohibited during the trial {11d}

The participants will have no study-related dietary restrictions. Hence, participants will consume carotenoids with their regular diet, and a periodic food frequency questionnaire will assess participants’ carotenoid intake. However, the study coordinator admonishes participants in both study arms to avoid carotenoid-containing supplements for the study duration.

### Provisions for post-trial care {30}

All participants who complete the study (i.e., undergo a final assessment within 2 weeks of giving birth) are given a 3-month supply of the supplement they were randomized for continued use.

### Outcomes {12}

This study’s primary objective is to precisely characterize mothers’ carotenoid status during pregnancy to determine if prenatal supplementation counteracts maternal systemic and ocular carotenoid depletion. The primary outcome will measure changes in participants’ carotenoid levels from enrollment to final study visit (birth of the child) as measured in the serum, skin, and macula by high-performance liquid chromatography (HPLC), resonance Raman spectroscopy, and dual-wavelength autofluorescence, respectively.

The secondary outcome will quantify maternal serum, skin, and ocular carotenoid levels at the end of each trimester and compare the systemic and ocular carotenoid status of infants whose mothers were in the Carotenoid group to those of the Control group.

The exploratory outcome will assess and compare maternal visual function (visual acuity and contrast sensitivity) throughout pregnancy and after birth between the two study arms. Furthermore, the exploratory outcome will evaluate the infant’s foveal structure at birth with spectral-domain optical coherence tomography (SD-OCT) and compare the parameters of infants whose mothers were in the Carotenoid group to those whose mothers were in the Control group.

### Participant timeline {13}

L-ZIP is expected to last for 2 to 3 years depending on the pace of enrollment. Table 1 details participants’ timeline and assessments for the study.

**Table 1:** Timeline and assessment schedule for the L-ZIP study

	Screening/baseline	Maternal Supplementation		
Visit Timeframe	T1	T2	T3	Birth
	Before 14 Wks. GA	22–26 Wks. GA	37–39 Wks. GA	0 to 2 Wks.
Informed consent	X			
Randomization	X			
Maternal visual acuity and contrast sensitivity	X	X	X	X
Maternal nutritional surveys	X	X	X	X
Dilated eye examination	X	X	X	X
Maternal serum carotenoids	X	X	X	X
Maternal macular pigment imaging	X	X	X	X
Maternal skin carotenoids	X	X	X	X
Dispense prenatal supplement and do pill counts	X	X	X	X
Infant cord blood carotenoids				X
Infant macular pigment imaging				X
Infant foveal imaging (SD-OCT)				X
Infant skin carotenoids				X

*T1*, first trimester; *T2*, second trimester; *T3*, third trimester; *Wks.*, weeks; *GA*, gestational age; *SD-OCT*, spectral-domain optical coherence tomography

### Sample size {14}

Statistical power and sample size for one of the primary outcome measures, maternal skin carotenoids at birth, was determined using data from the previous study by Henriksen et al. [32] in which the average maternal skin carotenoids ± standard deviation at birth was 34,000 ± 8300. This value is 20% lower than the University of Utah Moran Eye Center clinic average value of ~ 42,500 Raman counts reported for the ancillary AREDS 2 study [51]. With a power of 0.90 and an alpha level of 0.05, a sample size of 20 was calculated for each group to detect and prevent a 20% decline during pregnancy. In order to accommodate subsequent ineligibility due to premature birth, low birth weight, or other problems (~ 10%) and to plan for expected non-compliance and loss to follow-up (~ 20%), we intend to enroll 30 subjects in the Carotenoid group and another 30 in the Control group.

The other outcome measures (maternal ocular carotenoid status during pregnancy, infant carotenoid status at birth, and the exploratory outcome measures of foveal maturity at birth and visual function changes during pregnancy) are not as well characterized. However, we estimate that a similar sample size of 20 patients per group will be adequate to detect differences in these indices.

### Recruitment {15}

Participant recruitment for L-ZIP is mainly from the Obstetrics and Gynecology Department of the University of Utah Hospital. Hence, physicians in the Obstetrics and Gynecology Department are regularly informed about the trial and asked to help with recruitment. Moreover, the call for L-ZIP volunteers is published on John A. Moran’s Eye Center website, specifically on the clinical trial page. Potential and interested volunteers are invited to the John A. Moran Eye Center for screening to ascertain their eligibility. The screening assessments include obtaining demographic information, visual function measures, and complete ophthalmic examination. Of note, consultant ophthalmologists with retinal subspecialty undertake the comprehensive ophthalmic examination. Once eligibility is confirmed, participants provide informed consent to the IRB-approved study protocol before enrollment and study assessments.

## Assignment of interventions: allocation

### Sequence generation {16a}

The participants will be randomly assigned in a 1:1 ratio using a computer-generated random sequence to receive either the study intervention or placebo. The randomization is to ensure an equal assignment of participants to each study arm.

### Concealment mechanism {16b}

The Pharmacist at John A. Moran Eye Center, who has no contact with study subjects, assigns participants to study groups based on the randomization code list. The study team and participants receive only a bottle of softgels with the participant identification label.

### Implementation {16c}

L-ZIP is a double-masked trial; hence, both researchers and study participants are oblivious of study assignment. Participants will receive study formulations with only identification labels.

## Assignment of interventions: blinding

### Who will be blinded? {17a}

The study participants, clinicians, and research staff involved in all patient evaluations will be masked to the study treatment assignments. Further, the statistics team will carry out interim study analysis on masked study data.

### Procedure for unblinding if needed {17b}

In the event of a medical emergency where the knowledge of participant treatment by masked individuals (e.g., the subject or her physician) is required, the PI will have the ability to unmask the treatment assignment for a specific participant and share that information with the appropriate parties. Participants who have had their treatment assignment unmasked secondary to a severe or unexpected adverse event or medical emergency will no longer receive study treatment.

For regulatory reporting and if required by local regulations, the Sponsor-Investigator will unmask study treatment for all serious, unexpected adverse reactions related to the study drug.

## Data collection and management

### Plans for assessment and collection of outcomes {18a}

Once a participant is enrolled in the study, sociodemographic information, visual function, dietary questionnaire, and maternal skin, serum, and ocular carotenoid status will be assessed at each study visit (i.e., baseline, before 14 weeks gestational age [GA]; 22–26 weeks GA; 37–39 weeks GA; and 0–2 weeks after giving birth). Also, infants’ foveal architecture, skin, MP, and umbilical cord blood carotenoid assessments will be measured within the first 2 weeks of birth. Trained study staff will perform the various study assessments and will enter the various information into the subject case report forms (CRFs). The data collected will be entered into the Research Electronic Data Capture (REDCap) database maintained at the University of Utah with help from the Center for Clinical and Translational Science (CCTS). Data will be reviewed and checked regularly for quality and completeness using computerized and manual procedures. Access to the study database will be strictly by authorization. The study assessments are detailed below.

### Demographic and lifestyle questionnaire

The demographic and lifestyle information of participants include contact details (i.e., name, date of birth, phone number(s), place of residence, email address), education, ethnicity, occupation, height and weight (for body mass index [BMI] calculation), medical history, ocular medical history, smoking habits (history and frequency), and alcohol consumption (average intake per week and frequency).

### Visual function

#### Best-corrected visual acuity (BCVA)

BCVA assessment is by the logarithm of minimum angle of resolution (LogMAR) Early Treatment Diabetic Retinopathy Study (ETDRS) test charts viewed at 4 m using standardized protocols. The test charts consist of three distinct types (modified ETDRS charts 1, 2, and R), with a retro-illuminated box providing standardized chart illumination. The chart uses the Sloan ETDRS optotype for the BCVA test. Briefly, after the right eye test (with left eye occluded) is done, the right eye is occluded, and chart 2 replaces chart 1. The test is repeated for the left eye at the same distance. On completion of the left eye test, chart R replaces chart 2. Each letter read correctly is scored as 1 point, whereas incorrectly read is scored 0. The examiner records each row’s score, the total score, and the approximate Snellen Acuity Equivalents (determined based on the lowest row read with one or few mistakes) on the Visual Acuity Worksheet Form.

#### Contrast sensitivity (CS)

CS is measured using Pelli-Robson contrast sensitivity test charts viewed at 1 m using standardized protocols. The test charts consist of three distinct types (modified charts 1, 2, and R), with a retro-illuminated box providing standardized chart illumination. The participants read out loud the Sloan optotypes with the unoccluded eye. Of note, each eye assessment uses one of the modified test charts to avoid participants’ memorization of the optotypes. Each letter read correctly or incorrectly is scored (in log units) and recorded accordingly. The examiner records each row’s score and the total score (determined based on the lowest row read with one or few mistakes) on the Contrast Sensitivity Worksheet Form.

#### Skin carotenoid measurement

Skin carotenoid measurements in infants and their mothers are by resonance Raman spectroscopy devices. These validated noninvasive devices serve as biomarkers of fruit and vegetable intake that significantly correlate with skin and serum total carotenoid levels [52–55]. L-ZIP uses a high-sensitivity scanner suitable for measurements of infant and adult skin carotenoids. The Raman devices present little or no risk to the subjects. In brief, the measurements are taken by placing a probe on the subject’s palm (adults) or sole (infants) with a 488-nm blue laser light that does not generate heat and is harmless to one’s vision as long as it is not viewed directly for a long time (similar in risk to a laser pointer). The device collects back-scattered light and has a holographic notch filter that rejects Rayleigh-scattered light. The Peltier-cooled spectrograph analyzes the resulting fluorescence and Raman-shifted light. The peak intensity represents the C=C vibration of carotenoids at about 1525 cm^−1^ (known as Raman units [RU]). The device is calibrated daily, and the measurements require 30 s of skin contact and another 30 s to obtain the reading. Overall, three measurements (lasting for 3 min) will be taken, and the average used for statistical analysis.

### Macular pigment measurement

#### Pupillary dilation

Participants’ pupils will be dilated before carrying out macular pigment imaging. Thus, for mothers, a drop each of 1% tropicamide and 2.5% phenylephrine and, for infants, Cyclomydril™ drops will be used for dilatation. Notably, these eye drops are considered standard-of-care for dilatation during pregnancy and in newborns.

#### Maternal

Mothers’ macular pigment will be measured using the dual-wavelength autofluorescence method on the Heidelberg Multicolor Spectralis (Heidelberg Engineering GmbH, Heidelberg, Germany), which measures the attenuation of lipofuscin autofluorescence by blue absorbing macular pigment [56]. First, the examiner enters the participant details into the Heidelberg Eye Explorer (HEYEX version 1.7.1.0) software and asks the participant to fixate on a target with the dilated study eye. The fixation ensures good alignment and camera focus for quality retinal imaging. Autofluorescence images will be collected as the macula is raster-scanned sequentially with alternating 486 nm (blue) and 518 nm (green) lasers. Macular pigment images are prepared by digitally subtracting the green image from the blue image using appropriate correction factors to compensate for the absorption spectrum of the macular carotenoid pigment and then analyzed using a beta version of Heidelberg’s proprietary macular pigment analysis software after setting the zero point at 9° eccentricity from the point of fixation. We used 9° eccentricity to enable consistency and comparison with previous studies and to avoid the optic nerve and retinal blood vessels’ effect on MP measurements. Subsequently, MP measurements at 0.5°, 2°, and 9° eccentricities are recorded and used for statistical analysis. This instrument and software have proven highly reliable and reproducible [57–59], especially when measuring macular pigment optical volume at 9° eccentricity (MPOV 9°), which indicates the total of all MPOD values for all pixels with valid results within 9° eccentricity.

#### Infants

Infants’ macular measurements will be performed using blue light reflectometry [33, 60]. Under the supervision of a pediatric ophthalmologist, a certified ophthalmic photographer opens the swaddled baby’s eyes with a pediatric lid speculum. Posterior pole 80° images centered on the fovea will be taken with the RetCam (Natus, Pleasanton, CA, USA) retinal camera or equivalent using its optional blue light source and deliberately omitting the fluorescein angiography barrier filter in the collection light path. Color images will also be taken to facilitate the identification of crucial retinal landmarks.

#### Infant foveal anatomy

The Bioptigen SD-OCT (Leica Microsystems, Buffalo Grove, IL, USA) is an FDA-approved handheld portable unit used to image premature and full-term infants’ foveal anatomy. Swaddled infants will have their eyelids gently opened by the certified ophthalmic photographer or a pediatric ophthalmologist. A lid speculum to hold the eyelids and proparacaine to numb the eye may be used, if necessary, during imaging. Vertical and horizontal scans of the retina will then be acquired. The OCT images of the infant precede the contact images with the RetCam.

#### Serum carotenoid levels

Blood samples obtained from mothers and infant cord blood will be analyzed for serum carotenoid levels using well-established laboratory protocols that provide baseline separations of all common dietary carotenoids.

At each study visit, we use 6 mL blood collection tubes (BD Vacutainer K_2_EDTA; Becton, Dickinson and Company, NJ, USA) to collect blood samples following standard venipuncture techniques. The collection tubes are inverted few times to ensure mixing of the clot activator (K_2_EDTA). The blood samples are allowed to clot at room temperature for 30 min and then centrifuged for 10 min at 3000*g* using Sorvall™ ST 16 Centrifuge (ThermoFisher Scientific Inc., Waltham, MA, USA). After centrifugation, the serum (sample of interest) is separated from the whole blood, kept in a well-labeled storage tube, and stored at − 80 °C until further analysis.

For serum carotenoid extraction, ethanol containing 0.1% butylated hydroxytoluene will be added to 200 μL of serum to separate the proteins and followed by ethyl acetate for carotenoid extraction. The sample is then vortexed for 30 s and centrifuged at 2000*g* for 5 min. Further extraction with ethyl acetate will be carried out twice and then once with hexane. All the organic extracts obtained will be dried down under nitrogen gas. Further, the organic extracts are cleaned with hexane/methanol/distilled water, and the supernatant recovered and dried. The resulting residues will be resuspended in HPLC mobile phase (methanol: methyl tert-butyl ether [80:20, v/v]) and centrifuged at 2000*g* for 10 min. The supernatant will be analyzed using Agilent 1260 series HPLC (Agilent Technologies Inc., Santa Clara, CA, USA) on a C30 column (YMC Carotenoids, Allentown, PA, USA; 25 cm length × 4.6 mm internal diameter; maintained at room temperature) using diode array detection at a wavelength of 450 nm and a flow rate 1 mL/min for 50 min. Diode array spectra and co-elution with authentic standards identify and confirm the various carotenoid peaks.

### Dietary questionnaires

At each study visit, mothers complete the LZQ™ quantitative food frequency questionnaire (analyzed by Elizabeth Johnson, PhD, at Tufts University, Boston, MA, USA) that captures ~ 90% of the lutein/zeaxanthin foods consumed in the USA, based on the National Health and Nutrition Examination Survey (NHANES) data.

### Plans to promote participant retention and complete follow-up {18b}

Study follow-up visits coincide with participants’ routine prenatal visits to their obstetrician, at approximately 22–26 weeks GA, at 37–39 weeks GA, and within 2 weeks of giving birth. Before follow-up appointments, the study team will contact participants, either over the phone or through text messages, to enhance participant retention and study completion. Finally, participants will receive reimbursement for their time and transportation in the form of a gift card.

### Data management {19}

All study data will be entered into REDCap and will be accessible to only authorized personnel. Further, the study monitoring team from the University of Utah Compliance Office will review the database for accuracy and completeness.

### Confidentiality {27}

REDCap servers are encrypted, Health Insurance Portability and Accountability Act (HIPAA) compliant, password-protected, and accessible only by designated study members. Hard copy data collection forms will be stored in a locked cabinet with limited access only to designated members. Also, the statistical team will be given access to a de-identified dataset for interim statistical analyses.

### Plans for collection, laboratory evaluation, and storage of biological specimens for genetic or molecular analysis in this trial/future use {33}

A study staff trained in the protocol will carry out study assessments following standard procedures, and trained laboratory staff will process and analyze the samples following standard laboratory procedures. Biological specimens such as placenta tissue, saliva samples, and blood samples collected during the study will be kept in a tissue bank following standard protocols for future research. Participants’ identities will be coded so that samples cannot be traced to a specific person.

## Statistical methods

### Statistical methods for primary and secondary outcomes {20a}

Baseline characteristics between the Carotenoid and Control groups will be assessed for statistically significant differences using descriptive statistics. Independent sample *t*-tests (mean, standard deviation) will be used for continuous variables, while chi-square tests will be used for categorical variables. We expect to have no statistically significant differences in baseline characteristics in both groups because of the randomization process. Nevertheless, any between-group differences in baseline characteristics will be controlled for in subsequent analyses, as appropriate.

The primary, secondary, and exploratory efficacy analyses will be performed using the modified Intent-to-Treat subject set. Paired *t*-tests will compare the carotenoid status at enrollment with the final study visit of mothers receiving carotenoid supplementation to mothers not receiving carotenoid supplementation. We will also compare the carotenoid status of infants whose mothers received carotenoid supplementation with infants whose mothers received the placebo. Furthermore, repeated-measures analysis of variance (ANOVA) for all dependent variables will be conducted to determine whether there are changes in any of the dependent variables over the study period associated with the group. Time (T1 to postpartum) is regarded as the within-subject factor, while group (Carotenoid and Control) is considered as the between-subject factor. A time × group interaction will be conducted for all the dependent variables to determine whether changes in the dependent variables over the study period differ between the Carotenoid and Control groups. The associations between maternal and infant carotenoid status will be assessed with regression analysis. Statistical significance will be set at 2-tailed *p* < 0.05 for all analyses.

### Interim analyses {21b}

Masked data from participants who have completed the study visit will be used for the interim analysis. Thus, a linear mixed model will be deployed to assess the change in maternal systemic and ocular carotenoid status during pregnancy. Correlations between the various outcome measures of both mothers’ and infants’ carotenoid biomarkers will be assessed. Statistical significance will be set at *p* < 0.05 for all analyses.

### Methods for additional analyses (e.g., subgroup analyses) {20b}

Subgroup analyses will be performed to assess the influences of maternal dietary carotenoid intake, compliance with study supplement use, and co-existing medical conditions, BMI, infant gender, and other factors on our study results, with proper attention to the effects of multiple tests on the ability to draw a conclusion.

### Methods in analysis to handle protocol non-adherence and any statistical methods to handle missing data {20c}

A linear mixed model (with all the required adjustments) will be used to account for participants’ non-adherence and missing data.

### Plans to give access to the full protocol, participant-level data, and statistical code {31c}

The protocol of the study is publicly available on ClinicalTrials.gov identifier (NCT 03750968). The Principal Investigator will give access to the study de-identified dataset on reasonable request.

## Oversight and monitoring

### Composition of the coordinating center and trial steering committee {5d}

As earlier indicated, this study is a single-site RCT, and as such, the PI and the study team at the John A. Moran Eye Center will manage the trial. The trial steering committee, consisting of the data and safety monitoring committee (DSMC), PI, and the regulation committee, will ensure the accuracy and completeness of the study data. Notably, the committee will have overall responsibility and authority for directing activities, formulating policies for the study, and changing the study protocol.

### Composition of the data and safety monitoring committee, its role and reporting structure {21a}

The DSMC members will include the PI, an independent obstetrician, and a pediatric ophthalmologist. The selection of DSMC members will be based on clinical expertise, knowledge of clinical research methodology and regulations, and the absence of conflicts of interest. The DSMC will meet at least every 6 months (or as deemed appropriate by the chairperson) at a suitable and convenient time for all members. The DSMC will assist the Sponsor-Investigator in protecting the interests of study participants and assuring the integrity of study conduct and results.

### Adverse event (AE) reporting and harms {22}

The FDA approves the study formulations as GRAS, so no adverse effect is expected with the study intervention. However, any unfavorable and unintended sign, including a clinically significant abnormal laboratory finding, symptom, or disease temporally associated with the use of a study drug, whether considered in relation to the study drug, will be monitored and reported in detail in the patient source documents, from the signing of the ICF until the final visit. The PI will evaluate and treat/follow up with all AEs until symptoms or values return to normal. Further, the various regulatory bodies (IRB and FDA) will be informed of the various AEs and appropriate measures executed accordingly.

### Frequency and plans for auditing trial conduct {23}

The Regulatory Coordinator and Auditor from the ethics committee of the University of Utah will have monitoring visits every 3 months. The monitoring team will review study conduct and compliance with the study protocol, good clinical practice, standard operation procedure, and applicable regulatory requirements.

### Plans for communicating important protocol amendments to relevant parties (e.g., trial participants, ethical committees) {25}

Any amendments to the study protocol deemed necessary will be discussed between the PI and the DSMC. The investigator will not implement any changes to the protocol without prior review and documented approval from the IRB of an amendment, except where necessary to eliminate immediate hazards to study subjects.

### Dissemination plans {31a}

The study findings will be disseminated through peer-reviewed scientific journal publications and conference presentations. Moreover, study results will be communicated at relevant professional, clinical, and scientific meetings.

## Discussion

The L-ZIP study is designed to test the hypothesis that prenatal carotenoid supplementation will counteract maternal carotenoid depletion and improve systemic and ocular biomarkers of carotenoid status in the mother and the child. To the best of our knowledge, no prospective RCT has characterized maternal and infants’ ocular and systemic biomarkers of carotenoid status in healthy mothers throughout pregnancy. Hence, L-ZIP will provide relevant preliminary data for future, large-scale prospective clinical trials. A novel and crucial characteristic of the L-ZIP trial is the comprehensive measurement of MP, skin carotenoids, and mothers and infants’ visual function throughout pregnancy. Another unique feature worth mentioning is the use of L and Z in quantities comparable to AREDS2 as the study formulation [14].

Previous studies to ascertain carotenoid status in mother-infant pairs have been correlational. A recently published study by Thoene et al. investigates the association between placental L + Z and maternal serum L + Z, umbilical cord blood, and maternal dietary intake. The study reported L and Z as the most abundant carotenoid in the placenta and the umbilical cord blood. Also, L + Z in the placenta was significantly associated with umbilical cord blood and maternal serum L + Z, but not with maternal dietary intake. Of note, confounders that affect the carotenoid status, such as smoking and BMI, were not reported [29]. Furthermore, Henriksen et al. reported an interesting finding in their comparative study between mother-infant pairs [32]. The study found a significant association between infants’ macular pigment optical density (MPOD) and serum Z. Similarly, infants’ Z correlated with maternal serum Z [32]. Although these studies were not randomized controlled trials, their findings provide relevant information regarding carotenoid status in mothers and infants.

Although L-ZIP findings will help our understanding of maternal carotenoid status throughout pregnancy, it is worth pointing out that the study will not investigate carotenoids in maternal breastmilk. We think carotenoid content in breastmilk is a function of carotenoids in systemic circulation. Hence, adequate maternal systemic carotenoid levels (through diet or supplement intake) will invariably confer benefits to breastfed infants. A growing body of literature suggests that carotenoids enhance cognition [9, 23]; hence, a follow-up study to investigate infants’ cognitive function will improve our understanding of carotenoids’ role in cognitive development.

L-ZIP will assess through an adequately powered, prospective RCT the impact of prenatal maternal carotenoid supplementation on biomarkers of maternal and infants’ carotenoid status during pregnancy and how supplementation affects maternal visual function and infants’ foveal architecture. Subsequently, L-ZIP will inform and advance our understanding of carotenoids’ protective effects during pregnancy.

## Trial status

Trial recruitment began on September 26, 2019, and is still open for recruitment under protocol version 4.0 dated October 9, 2019. Presently, enrollment is 70% complete (42/60), and the enrollment completion has been extended to April 30, 2021, due to COVID-related delays.

## Abbreviations

ANOVA: Analysis of variance
AREDS: Age-Related Eye Disease Study
BCVA: Best-corrected visual acuity
BMI: Body mass index
CCTS: Center for Clinical and Translational Science
CRF: Case report form
CS: Contrast sensitivity
DSMC: Data and safety monitoring committee
ETDRS: Early Treatment Diabetes Retinopathy Study
FDA: Food and Drug Administration
GA: Gestational age
GRAS: Generally Recognized as Safe
HIPAA: Health Insurance Portability and Accountability Act
HPLC: High-performance liquid chromatography
ICF: Informed consent form
ICH: International Conference of Harmonization
L: Lutein
LogMAR: Logarithm of minimum angle of resolution
L-ZIP: Lutein and Zeaxanthin in Pregnancy
MacTel: Macular telangiectasia type II
MP: Macular pigment
MZ: *meso-*Zeaxanthin
NHANES: National Health and Nutrition Examination Survey
PI: Principal Investigator
RCT: Randomized controlled trial
REDCap: Research Electronic Data Capture
SD-OCT: Spectral-domain optical coherence tomography
SPIRIT: Standard Protocol Items: Recommendations for Interventional Trials
Z: Zeaxanthin

## References

[1] Arunkumar R, Calvo CM, Conrady CD, Bernstein PS (2018). What do we know about the macular pigment in AMD: the past, the present, and the future. Eye (London, England).

[2] Bone RA, Landrum JT, Tarsis SL (1985). Preliminary identification of the human macular pigment. Vis Res.

[3] Khachik F, Bernstein PS, Garland DL (1997). Identification of lutein and zeaxanthin oxidation products in human and monkey retinas. Invest Ophthalmol Vis Sci.

[4] Hirsch J, Curcio CA (1989). The spatial resolution capacity of human foveal retina. Vis Res.

[5] Snodderly DM, Brown PK, Delori FC, Auran JD (1984). The macular pigment. I. Absorbance spectra, localization, and discrimination from other yellow pigments in primate retinas. Invest Ophthalmol Vis Sci.

[6] Bernstein PS, Li B, Vachali PP, Gorusupudi A, Shyam R, Henriksen BS, Nolan JM (2016). Lutein, zeaxanthin, and meso-zeaxanthin: the basic and clinical science underlying carotenoid-based nutritional interventions against ocular disease. Prog Retin Eye Res.

[7] Li B, Ahmed F, Bernstein PS (2010). Studies on the singlet oxygen scavenging mechanism of human macular pigment. Arch Biochem Biophys.

[8] Li SY, Fung FK, Fu ZJ, Wong D, Chan HH, Lo AC (2012). Anti-inflammatory effects of lutein in retinal ischemic/hypoxic injury: in vivo and in vitro studies. Invest Ophthalmol Vis Sci.

[9] Vishwanathan R, Iannaccone A, Scott TM, Kritchevsky SB, Jennings BJ, Carboni G, Forma G, Satterfield S, Harris T, Johnson KC, Schalch W, Renzi LM, Rosano C, Johnson EJ (2014). Macular pigment optical density is related to cognitive function in older people. Age Ageing.

[10] Akuffo KO, Nolan JM, Peto T, Stack J, Leung I, Corcoran L, Beatty S (2017). Relationship between macular pigment and visual function in subjects with early age-related macular degeneration. Br J Ophthalmol.

[11] Bernstein PS, Zhao DY, Wintch SW, Ermakov IV, McClane RW, Gellermann W (2002). Resonance Raman measurement of macular carotenoids in normal subjects and in age-related macular degeneration patients. Ophthalmology.

[12] Seddon JM, Ajani UA, Sperduto RD, Hiller R, Blair N, Burton TC, Farber MD, Gragoudas ES, Haller J, Miller DT (1994). Dietary carotenoids, vitamins A, C, and E, and advanced age-related macular degeneration. Eye Disease Case-Control Study Group. JAMA.

[13] Eye Disease Case-Control Study Group (1993). Antioxidant status and neovascular age-related macular degeneration. Arch Ophthalmol.

[14] Chew EY, Clemons TE, Sangiovanni JP, Danis RP, Ferris FL, Age-Related Eye Disease Study 2 Research Group (2014). Secondary analyses of the effects of lutein/zeaxanthin on age-related macular degeneration progression: AREDS2 report No. 3. JAMA Ophthalmol.

[15] SanGiovanni JP, Chew EY, Clemons TE, Ferris FL, Gensler G, Age-Related Eye Disease Study Research Group (2007). The relationship of dietary carotenoid and vitamin A, E, and C intake with age-related macular degeneration in a case-control study: AREDS Report No. 22. Arch Ophthalmol.

[16] Davinelli S, Ali S, Solfrizzi V, Scapagnini G, Corbi G (2021). Carotenoids and cognitive outcomes: a meta-analysis of randomized intervention trials. Antioxidants.

[17] Nouchi R, Suiko T, Kimura E, Takenaka H, Murakoshi M, Uchiyama A (2020). Effects of lutein and astaxanthin intake on the improvement of cognitive functions among healthy adults: a systematic review of randomized controlled trials. Nutrients.

[18] Scarmo S, Cartmel B, Lin H, Leffell DJ, Welch E, Bhosale P, Bernstein PS, Mayne ST (2010). Significant correlations of dermal total carotenoids and dermal lycopene with their respective plasma levels in healthy adults. Arch Biochem Biophys.

[19] Scott KJ, Thurnham DI, Hart DJ, Bingham SA, Day K (1996). The correlation between the intake of lutein, lycopene and beta-carotene from vegetables and fruits, and blood plasma concentrations in a group of women aged 50-65 years in the UK. Br J Nutr.

[20] Zimmer JP, Hammond BR (2007). Possible influences of lutein and zeaxanthin on the developing retina. Clin Ophthalmol.

[21] Bhosale P, Serban B, Zhao DY, Bernstein PS (2007). Identification and metabolic transformations of carotenoids in ocular tissues of the Japanese quail Coturnix japonica. Biochemistry.

[22] Johnson EJ, Neuringer M, Russell RM, Schalch W, Snodderly DM (2005). Nutritional manipulation of primate retinas, III: effects of lutein or zeaxanthin supplementation on adipose tissue and retina of xanthophyll-free monkeys. Invest Ophthalmol Vis Sci.

[23] Shyam R, Gorusupudi A, Nelson K, Horvath MP, Bernstein PS (2017). RPE65 has an additional function as the lutein to meso-zeaxanthin isomerase in the vertebrate eye. Proc Natl Acad Sci U S A.

[24] Malone JI (1975). Vitamin passage across the placenta. Clin Perinatol.

[25] Dimenstein R, Trugo NMF, Donangelo CM, Trugo LC, Anastácio AS (1996). Effect of subadequate maternal vitamin-a status on placental transfer of retinol and beta-carotene to the human fetus. Neonatology.

[26] Kvansakul J, Rodriguez-Carmona M, Edgar DF, Barker FM, Köpcke W, Schalch W (2006). Supplementation with the carotenoids lutein or zeaxanthin improves human visual performance. Ophthalmic Physiol Opt.

[27] Stringham JM, Hammond BR (2008). Macular pigment and visual performance under glare conditions. Optom Vis Sci.

[28] Johnson EJ (2014). Role of lutein and zeaxanthin in visual and cognitive function throughout the lifespan. Nutr Rev.

[29] Thoene M, Anderson-Berry A, Van Ormer M, Furtado J, Soliman GA, Goldner W (2019). Quantification of lutein + zeaxanthin presence in human placenta and correlations with blood levels and maternal dietary intake. Nutrients.

[30] Lieblein-Boff JC, Johnson EJ, Kennedy AD, Lai CS, Kuchan MJ (2015). Exploratory metabolomic analyses reveal compounds correlated with lutein concentration in frontal cortex, hippocampus, and occipital cortex of human infant brain. PLoS One.

[31] Vishwanathan R, Kuchan MJ, Sen S, Johnson EJ (2014). Lutein and preterm infants with decreased concentrations of brain carotenoids. J Pediatr Gastroenterol Nutr.

[32] Henriksen BS, Chan G, Hoffman RO, Sharifzadeh M, Ermakov IV, Gellermann W, Bernstein PS (2013). Interrelationships between maternal carotenoid status and newborn infant macular pigment optical density and carotenoid status. Invest Ophthalmol Vis Sci.

[33] Bernstein PS, Sharifzadeh M, Liu A, Ermakov I, Nelson K, Sheng X, Panish C, Carlstrom B, Hoffman RO, Gellermann W (2013). Blue-light reflectance imaging of macular pigment in infants and children. Invest Ophthalmol Vis Sci.

[34] Berti C, Cetin I, Agostoni C, Desoye G, Devlieger R, Emmett PM, Ensenauer R, Hauner H, Herrera E, Hoesli I, Krauss-Etschmann S, Olsen SF, Schaefer-Graf U, Schiessl B, Symonds ME, Koletzko B (2016). Pregnancy and infants’ outcome: nutritional and metabolic implications. Crit Rev Food Sci Nutr.

[35] Scaife AR, McNeill G, Campbell DM, Martindale S, Devereux G, Seaton A (2006). Maternal intake of antioxidant vitamins in pregnancy in relation to maternal and fetal plasma levels at delivery. Br J Nutr.

[36] Johnson EJ, Maras JE, Rasmussen HM, Tucker KL (2010). Intake of lutein and zeaxanthin differ with age, sex, and ethnicity. J Am Diet Assoc.

[37] Mathews F, Yudkin P, Smith RF, Neil A (2000). Nutrient intakes during pregnancy: the influence of smoking status and age. J Epidemiol Community Health.

[38] Thorne-Lyman AL, Fawzi WW (2012). Vitamin A and carotenoids during pregnancy and maternal, neonatal and infant health outcomes: a systematic review and meta-analysis. Paediatr Perinat Epidemiol.

[39] Abdel-Aal E-SM, Akhtar H, Zaheer K, Ali R (2013). Dietary sources of lutein and zeaxanthin carotenoids and their role in eye health. Nutrients.

[40] Pereira AC, Martel F (2014). Oxidative stress in pregnancy and fertility pathologies. Cell Biol Toxicol.

[41] Lorenzoni F, Giampietri M, Ferri G, Lunardi S, Madrigali V, Battini L, Boldrini A, Ghirri P (2013). Lutein administration to pregnant women with gestational diabetes mellitus is associated to a decrease of oxidative stress in newborns. Gynecol Endocrinol.

[42] Sharma JB, Sharma A, Bahadur A, Vimala N, Satyam A, Mittal S (2006). Oxidative stress markers and antioxidant levels in normal pregnancy and pre-eclampsia. Int J Gynecol Obstet.

[43] Dutta-Roy AK (2000). Transport mechanisms for long-chain polyunsaturated fatty acids in the human placenta. Am J Clin Nutr.

[44] Carlson SE, Colombo J, Gajewski BJ, Gustafson KM, Mundy D, Yeast J, Georgieff MK, Markley LA, Kerling EH, Shaddy DJ (2013). DHA supplementation and pregnancy outcomes. Am J Clin Nutr.

[45] Crider KS, Cordero AM, Qi YP, Mulinare J, Dowling NF, Berry RJ (2013). Prenatal folic acid and risk of asthma in children: a systematic review and meta-analysis. Am J Clin Nutr.

[46] Ramakrishnan U, Gonzalez-Casanova I, Schnaas L, DiGirolamo A, Quezada AD, Pallo BC, Hao W, Neufeld LM, Rivera JA, Stein AD, Martorell R (2016). Prenatal supplementation with DHA improves attention at 5 y of age: a randomized controlled trial. Am J Clin Nutr.

[47] Tamura T, Picciano MF (2006). Folate and human reproduction. Am J Clin Nutr.

[48] Kiely M, Cogan PF, Kearney PJ, Morrissey PA (1999). Concentrations of tocopherols and carotenoids in maternal and cord blood plasma. Eur J Clin Nutr.

[49] Oostenbrug GS, Mensink RP, Al MD, van Houwelingen AC, Hornstra G (1998). Maternal and neonatal plasma antioxidant levels in normal pregnancy, and the relationship with fatty acid unsaturation. Br J Nutr.

[50] Zhang C, Williams MA, Sanchez SE, King IB, Ware-Jauregui S, Larrabure G, Bazul V, Leisenring WM (2001). Plasma concentrations of carotenoids, retinol, and tocopherols in preeclamptic and normotensive pregnant women. Am J Epidemiol.

[51] Bernstein PS, Ahmed F, Liu A, Allman S, Sheng X, Sharifzadeh M, Ermakov I, Gellermann W (2012). Macular pigment imaging in AREDS2 participants: an ancillary study of AREDS2 subjects enrolled at the Moran Eye Center. Invest Ophthalmol Vis Sci.

[52] Ermakov IV, Ermakova MR, Bernstein PS, Chan GM, Gellermann W (2013). Resonance Raman based skin carotenoid measurements in newborns and infants. J Biophotonics.

[53] Ermakov IV, Gellermann W (2010). Validation model for Raman based skin carotenoid detection. Arch Biochem Biophys.

[54] Ermakov IV, Whigham LD, Redelfs AH, Jahns L, Stookey J, Bernstein PS (2016). Skin carotenoids as biomarker for vegetable and fruit intake: validation of the reflection-spectroscopy based “Veggie Meter”. FASEB J.

[55] Mayne ST, Cartmel B, Scarmo S, Jahns L, Ermakov IV, Gellermann W (2013). Resonance Raman spectroscopic evaluation of skin carotenoids as a biomarker of carotenoid status for human studies. Arch Biochem Biophys.

[56] Akuffo KO, Beatty S, Stack J, Peto T, Leung I, Corcoran L, Power R, Nolan JM (2015). Concordance of macular pigment measurement using customized heterochromatic flicker photometry and fundus autofluorescence in age-related macular degeneration. Invest Ophthalmol Vis Sci.

[57] Conrady CD, Bell JP, Besch BM, Gorusupudi A, Farnsworth K, Ermakov I, Sharifzadeh M, Ermakova M, Gellermann W, Bernstein PS (2017). Correlations between macular, skin, and serum carotenoids. Invest Ophthalmol Vis Sci.

[58] Green-Gomez M, Bernstein PS, Curcio CA, Moran R, Roche W, Nolan JM (2019). Standardizing the assessment of macular pigment using a dual-wavelength autofluorescence technique. Transl Vis Sci Technol.

[59] You QS, Bartsch DU, Espina M, Alam M, Camacho N, Mendoza N (2016). Reproducibility of macular pigment optical density measurement by two-wavelength autofluorescence in a clinical setting. Retina (Philadelphia, Pa).

[60] Sharifzadeh M, Bernstein PS, Gellermann W (2013). Reflection-based imaging of macular pigment distributions in infants and children. J Biomed Opt.

